# Apology

**Published:** 1881-08-20

**Authors:** 


### EDITORIALS AND MISCELLANEOUS.

### *APOLOGY.*

The delay in the present issue of THE RECORD was occasioned by a move of our printing establishment to a new and better place. Our readers will please excuse us.

